# Miscellaneous Items

**Published:** 1886-10

**Authors:** 


### MISCELLANEOUS ITEMS.

**Stopping Hiccough.**—A Brazilian physician, states that refrigeration of the lobe of the ear will stop hiccough, whatever its cause may be. Very slight refrigeration will answer, the application of cold water or even saliva being sufficient.

**Three hundred thousand pounds** of snuff are sold annually in Atlanta, Ga., according to the *Surg. Jour.* of that place. It is all consumed in that section and not exported, and that city is said to be the third largest snuff market in the world.

**The following liniment** is recommended for earache:—"Camphorated chloral 2½ parts, pure glycerine 16½ parts, and oil of sweet almonds 10 parts." This is to be well mixed and preserved in an hermetically sealed bottle. Applications may be made by means of a pledget of soft cotton soaked in the liniment and put in the ear twice a day.

**Catarrhal Headache.**—Iodide of potassium is said to quickly relieve the dull headache so often accompanying an ordinary cold in the head. Two grains may be dissolved in a glass full of water, which is to be taken in little sips during half an hour. Dr. Davis recommends this simple remedy, and says he has hardly ever known it to fail.

**A Bad Breath** may be cured as follows, no matter what the cause: Three hours after breakfast a teaspoonful of the following mixture: Chlorate of potassa, two drachms; sweetened water, four ounces; wash the mouth occasionally with a similar mixture, and the breath will be as sweet as an infant's of two months.

**A girl** was taken before the Paris tribunal charged with stealing a blanket. She pleaded that she was under the influence of another person and could not help herself. In prison it was found that she was in a hypnotized condition, and acted readily under the commands of others, doing anything that was told her. She was examined by a commission of Charcot, Brouardel, and Mollet, who reported that this state came from the use of morphia, suffering, and hunger. That these suggestions from others, acting on an unstable nervous organism, greatly deranged by morphia and other causes, rendered her irresponsible for her acts. She was acquitted.

**Toxic Idiocy.**—In the the children of alcohol and opium inebriates is far more frequent than is supposed. In the history of twenty cases taken indiscriminately, eight were found to come from inebriate parents. Quite a large percentage of these cases come from the use of opium and beer in infancy. The former in the shape of soothing syrup to quiet children who are irritable, and the latter to give them strength. In these cases some state of atrophy of the nerve-centres takes place, and arrests of development from faulty nutrition. In a neurotic family this is a source of great danger.—*Q. J. of Inebriety.*

**The ten commandments** for bathers: 1. Do not bathe when excited. 2. Do not bathe when feeling badly. 3. Do not bathe after having been up all night or after excessive exertion, before resting several hours. 4. Do not bathe after having taken a heavy meal or alcoholic drinks. 5. Walk slowly to the bathing place. 6. Inquire after the depth and the current of the water as soon as you arrive there. 7. Undress slowly, but then go into the water at once. 8. Jump into the water with your head first or wet the head quickly if you cannot do the first. 9. Do not remain in the water long, especially if you are not very strong. 10. After the bath rub the body well to aid circulation of the blood and take moderate exercise. Bathing and swimming is useful for body and soul, not alone in warm but also in cool weather, if above advice is heeded.

**The Normal Man.**—Huxley asserts that the proper weight of man is 154 pounds, made up as follows: Muscles and their appurtenances, 68 pounds; skeleton, 24 pounds; skin, 10½ pounds; fat, 28 pounds; brain, 3 pounds; thoracic viscera, 3½ pounds; abdominal viscera, 11 pounds; blood which would drain from the body, 7 pounds. The heart of such a man should beat 75 times a minute, and he should breathe 15 times a minute. In 24 hours he should vitiate 1,750 cubic feet of pure air to the extent of 1 per cent. He would throw off by the skin 18 ounces of water, 300 grains of solid matter, and 400 grains of carbonic acid every 24 hours; and his total loss during that period would be 6 pounds of water and a little more than 20 pounds of other matter.

**Signs of the Tongue.**—The tongue is the indicator of the system. A white-coated tongue indicates febrile disturbance; a brown, moist tongue indicates disordered digestion or overloaded *primæ viæ;* a brown, dry tongue indicates depressed vitality, as in typhoid conditions and blood poisoning; a red, moist tongue indicates debility, as from exhausting discharges; a red, dry tongue indicates pyrexia, or any inflammatory fever; a "strawberry" tongue, with prominent papillæ, indicates scarlet fever or rotheln; a red, glazed tongue indicates debility, with want of assimilative power of digestion; a tremulous, flabby tongue indicates delirium tremens; hesitancy in protruding the tongue indicates concussion of the brain; protrusion at one side indicates paralysis of the muscles on that side.

**A gentleman** received a note from his lawyer which he was unable to decipher. On his way to his office he met a friend at the door of a drug store. The friend, after vainly attempting to read the note, suggested that they step inside and hand it to the druggist without comment. The druggist, after studying it in silence for a few minutes, stepped behind his prescription case, and in a short time returned with a bottle of medicine, duly labeled and bearing directions. When the gentleman saw his lawyer, he was informed that the note was a notice for him to call at his office between three and four P. M. of the following day. It is a pretty difficult matter to "stick" the regulation druggist.

**An exchange** tells an amusing story of a modest young man, who was employed on his own proposition, to serve in a counting room, on a salary of one cent a month to be doubled every succeeding month for three years. His employer thought if he were ever so great a dunce he could get that worth of work out of him, but after making the contract, before the first half year year had passed, he was glad to compromise with his stupid assistant at $5,000. Here are the figures: First month, .01; second, .02; third, .04; fourth, .08; fifth, .16; sixth, .32; seventh, .64; eight, $1 28; ninth, $2.56; tenth, $5.12; eleventh, $10.24; twelfth, $20.48; thirteenth, $40.96; fourteenth, $81.92; fifteenth, $163.84; sixteenth, $327.68; seventeenth, $655.36; eighteenth, $1,311.72; nineteenth, $2,623.44; twentieth, $5,246.88; twenty-first, $10,493.75; twenty-second, $20,986.52; twenty-third, $41,975.04; twenty-fourth, $83,950.08; twenty-fifth, $167,900.16; twenty-sixth, $335,800.32; twenty-seventh, $671,600.64; twenty-eighth, $1,333,210.28; twenty-ninth, $2,686,402.56; thirtieth; $5,372,804.12; thirty-first, $10,745,610.24; thirty-second, $21,491,220.48 thirty-third, $42,982,440.96; thirty-fourth, $85,964,881.92; thirty-fifth, $171,929,653.84; thirty-sixth, $343,858,529.68.

### *PILL OR THRILL.*

Said Dr. Pill to Dr. Thrill,
'Tis rumored Jones is sorely ill.
    'Tis even said
    He's out of his head,
Do tell me, is it true?

If Jones is ill, said Dr. Thrill,
We've but to wait to know his will.
    If *sane*, you'll see,
    He'll send foı me;
If out of his head, for *you*.

